# Photocatalytic Evaluation of Fe_2_O_3_–TiO_2_ Nanocomposites: Influence of TiO_2_ Content on Their Structure and Activity

**DOI:** 10.3390/molecules30214309

**Published:** 2025-11-05

**Authors:** Israel Águila-Martínez, Pablo Eduardo Cardoso-Avila, Isaac Zarazúa, Héctor Pérez Ladrón de Guevara, José Antonio Pérez-Tavares, Efrén González-Aguiñaga, Rita Patakfalvi

**Affiliations:** 1 Centro Universitario de los Lagos, Universidad de Guadalajara, Lagos de Moreno 47460, Jalisco, Mexico; ing.bioq.iam@gmail.com (I.Á.-M.); isaac.zarazua@academicos.udg.mx (I.Z.); hector.pladrondeguevara@academicos.udg.mx (H.P.L.d.G.); jose.perez5161@academicos.udg.mx (J.A.P.-T.); egonzaleza@enes.unam.mx (E.G.-A.); 2 Centro de Investigaciones en Óptica, A.C., León 37150, Guanajuato, Mexico; pecardoso@cio.mx

**Keywords:** Fe_2_O_3_–TiO_2_ composites, solvothermal synthesis, photocatalysis, methylene blue degradation, band gap engineering

## Abstract

In this study, Fe_2_O_3_–TiO_2_ nanocomposites with different TiO_2_ contents (1–50%) were synthesized via a solvothermal method using pre-formed α-Fe_2_O_3_ nanoparticles as cores. We systematically evaluated the influence of TiO_2_ loading on the nanocomposites’ structural, morphological, optical, and photocatalytic properties. X-ray diffraction revealed the coexistence of hematite and anatase phases, with an increase in TiO_2_ content inducing reduced crystallite size, enhanced dislocation density, and microstrain, indicating interfacial lattice distortion. Scanning electron microscopy (SEM) and energy-dispersive X-ray spectroscopy (EDS) showed a uniform elemental distribution at low TiO_2_ contents, evolving into irregular agglomerates at higher loadings. Fourier-transform infrared (FTIR) spectra indicated the suppression of Fe–O vibrations and the appearance of hydroxyl-related bands with TiO_2_ enrichment. Diffuse reflectance spectroscopy (DRS) analysis confirmed the simultaneous presence of hematite (~2.0 eV) and anatase (3.2–3.35 eV) absorption edges, with a slight blue shift in the TiO_2_ band gap at higher concentrations. Photocatalytic activity, assessed using methylene blue degradation under xenon lamp irradiation, demonstrated a strong dependence on the TiO_2_ fraction. The composite containing 33% TiO_2_ achieved the best performance, with 98% dye removal and a pseudo-first-order rate constant of 0.045 min^−1^, outperforming both pure hematite and commercial P25 TiO_2_. These results highlight that intermediate TiO_2_ content (~33%) provides an optimal balance between structural integrity and photocatalytic efficiency, making Fe_2_O_3_–TiO_2_ heterostructures promising candidates for water purification under simulated solar irradiation.

## 1. Introduction

Synthetic organic dyes are widely used as coloring substances in the textile, tannery, paper, pharmaceutical, cosmetics and food industries [1]; however, due to their large-scale production and widespread applications, these dyes can cause serious environmental impacts when released untreated into natural ecosystems. They resist biodegradation, causing pollution of soil and aquatic environments [2].

Various approaches are used to treat dye-contaminated wastewater, encompassing physical methods, such as adsorption, ion exchange, and membrane filtration; chemical methods, such as coagulation–flocculation, electrochemical methods, and advanced oxidation processes; and biological methods, such as enzyme-, bacteria-, fungal-, yeast-, or algae-assisted degradation [3].

In this regard, advanced oxidation processes are potentially greener methods, as they generate less waste than other available methods [1,4].

Iron oxide-based nanomaterials have also been widely studied for environmental remediation, with approaches ranging from adsorption to photocatalysis [5,6]. Of these photocatalysts, TiO_2_ has received significant attention due to its chemical stability, low cost, and strong oxidizing power under UV illumination [7,8]. However, its wide band gap (~3.2 eV in anatase) limits its efficiency under visible light, which restricts its practical application in solar-driven processes [9]. On the other hand, hematite (α-Fe_2_O_3_), a narrow-band gap semiconductor (~2.0 eV), offers visible-light absorption but suffers from rapid electron–hole recombination and poor photoconductivity [10].

To overcome these limitations, titania (TiO_2_) and hematite (α-Fe_2_O_3_) have been combined into a binary nanocomposite to enhance visible-light activity, improve charge separation, and exploit the synergy between both oxides; this approach has shown promise [11,12,13]. Such heterostructures can exhibit improved photocatalytic performance, as their interfacial interactions promote charge transfer and broaden the spectral response [14].

The abovementioned combination has been demonstrated to enhance photocatalytic efficiency by coupling the visible-light absorption of Fe_2_O_3_ with the high chemical stability and conduction-band potential of TiO_2_. Various synthetic strategies—such as sol–gel, hy-drothermal, and solvothermal routes—have been used to control crystallite size, interface quality, and phase composition, leading to significant variations in photocatalytic performance. Fe_2_O_3_–TiO_2_ composites have been reported to exhibit superior activity compared with pristine oxides, mainly due to interfacial interactions that may facilitate charge separation and hinder recombination [15,16,17,18,19,20,21,22,23].

Despite these developments, the role of the TiO_2_ fraction as a tunable variable that governs the structure–activity relationship of Fe_2_O_3_–TiO_2_ nanocomposites remains insufficiently understood. Most reports have investigated a limited range of compositions or relied on different synthesis conditions, leaving it unclear how TiO_2_ content quantitatively affects crystallite size, optical properties, and photocatalytic kinetics. Further research is required on the systematic optimization and creation of a unified preparation method for this compositional parameter.

In this work, we address this research gap by systematically optimizing the TiO_2_ fraction (1–50%) in Fe_2_O_3_–TiO_2_ nanocomposites synthesized via a single solvothermal method, with the aim of correlating composition, structure, and photocatalytic activity. The mate-rials were characterized using X-ray diffraction (XRD), Fourier-transform infrared spectroscopy (FTIR), and scanning electron microscopy with energy-dispersive X-ray spectroscopy (SEM–EDS), while their optical and photocatalytic behaviors were evaluated through diffuse reflectance spectroscopy (DRS) and methylene blue degradation under simulated solar light. This composition-driven approach revealed the existence of an optimal TiO_2_ loading (~33%) that maximizes photocatalytic efficiency, offering a coherent framework to understand how interfacial balance, rather than mere phase combination, dictates performance. Our findings complement recent studies on Fe_2_O_3_–TiO_2_ coupling for visible-light photocatalysis [24] and provide new insight into the rational design of oxide-based photocatalysts.

## 2. Results and Discussion

### 2.1. X-Ray Diffraction Analysis and Structural Defects

**Figure 1:**
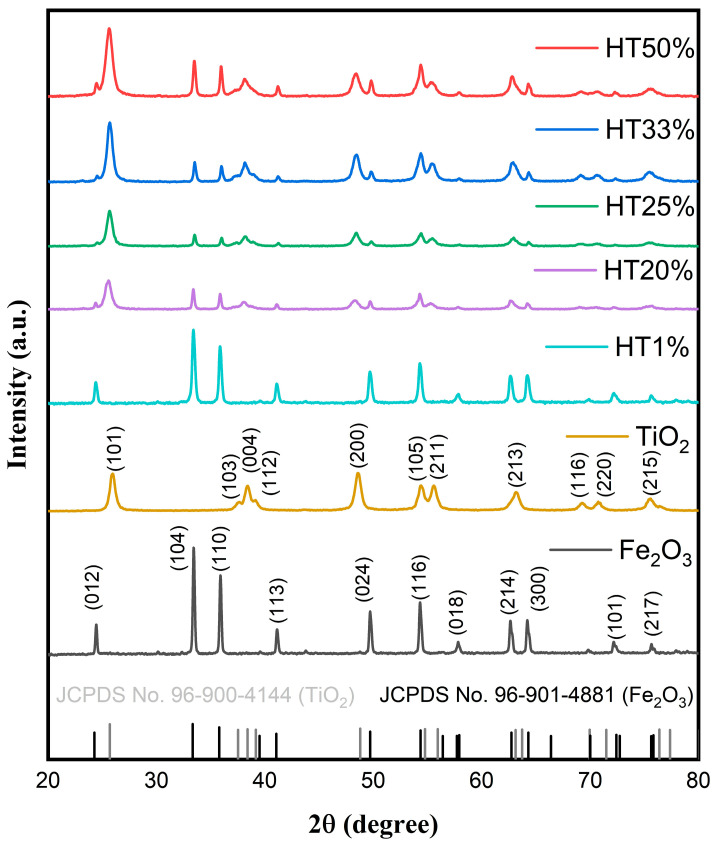
XRD patterns of pure Fe_2_O_3_, TiO_2_, and Fe_2_O_3_–TiO_2_ composites with different TiO_2_ contents, showing the coexistence of α-Fe_2_O_3_ and anatase TiO_2_ phases. Reference patterns from JCPDS cards for hematite (96-901-4881, in black) and anatase (96-900-4144, in gray) are also included for phase identification.

The crystalline structure of the Fe_2_O_3_–TiO_2_ binary composites was characterized using X-ray diffraction, with a 2θ range of 20–80°. Figure 1 shows the diffraction pattern of the representative composites. The XRD patterns clearly reveal characteristic diffraction peaks corresponding simultaneously to hematite (α-Fe_2_O_3_) and anatase-phase TiO_2_, confirming their coexistence without the formation of new detectable crystalline phases.

Characteristic diffraction peaks for hematite were observed at 24.31° (012), 33.24° (104), 35.75° (110), 41.12° (113), 49.71° (024), 54.24° (116), 57.75° (018), 62.55° (214), 64.09° (300), 75.57° (220), and 72.11° (101), matching with the rhombohedral α-Fe_2_O_3_ phase (space group R-3c, JCPDS Card No. 96-901-4881) [25]. Characteristic anatase TiO_2_ peaks were identified at 25.69° (101), 37.53° (103), 38.3° (004), 48.7° (200), 54.7° (105), 55.9° (211), 63.1° (213), 69.9° (116), and 75.3° (107), corresponding to the tetragonal anatase phase (space group I4_1_/AMD, JCPDS Card No. 96-900-4144) [26].

No additional peaks were detected, indicating the absence of secondary phases or new crystalline species resulting from Fe_2_O_3_–TiO_2_ interactions [15]. Partial peak overlapping between the hematite and anatase phases was observed due to structural similarities in their crystal planes, influencing their relative peak intensities but confirming their overall structural stability [16]. As the TiO_2_ content increased, the intensity of anatase diffraction peaks progressively increased, particularly for the (101), (004), and (200) planes. In contrast, the relative intensity of hematite peaks, such as (104) and (110), gradually decreased, reflecting the compositional shift within the binary system. These changes are consistent with the increasing proportion of TiO_2_ in the composites and confirm the successful incorporation of anatase without forming secondary phases. The coexistence of both phases was maintained across all compositions, and no peak shifting was detected, indicating structural stability and the absence of solid-state reactions between the oxides.

Microstructural parameters, including the average crystallite size (D), dislocation density (δ), microstrain (ε), and crystallinity percentage (Xc), were calculated and are summarized in Table 1. The average crystallite size was obtained using the Scherrer equation $D=\frac{K\lambda }{\beta cos\theta }$, where *K* is the shape factor (0.9), *λ* the Cu Kα wavelength (1.5406 Å), *β* the full width at half maximum (FWHM) of the diffraction peak (in radians), and *θ* the Bragg angle. The dislocation density was estimated using the equation $\delta =\frac{1}{D^{2}}$, and microstrain was calculated using the Williamson–Hall method $\beta cos\theta =\frac{K\lambda }{D}+4\epsilon \;sin\theta$ [27]. The degree of crystallinity (Xc) was determined by separating the integrated areas of crystalline diffraction peaks from the total diffracted area, following the classical approach of Patterson [28].

For the hematite-rich material (HT1%), the α-Fe_2_O_3_ crystallites reached ~20 nm in size, accompanied by moderate dislocation density (2.55 × 10^14^ m^−2^) and microstrain (4.36 × 10^−3^), consistent with relatively ordered domains. Upon incorporating TiO_2_ (HT20–25%), the α-Fe_2_O_3_ crystallites were slightly enlarged (21–25 nm), while anatase was nucleated into ultrasmall domains (~10–11 nm) with remarkably high defect densities (δ ≈ 1.0 × 10^15^ m^−2^; ε ≈ 0.009–0.010). In the material with intermediate composition (HT33%), the hematite crystallite size decreased drastically (15.8 nm), while both δ and ε peaked (6.6 × 10^14^ m^−2^ and 4.7 × 10^−3^, respectively), indicating enhanced structural distortion.

It is important to note that TiO_2_ does not substitute into a hematite lattice, as the α-Fe_2_O_3_ phase is pre-formed prior to solvothermal TiO_2_ deposition. Instead, the observed microstructural evolution is attributed to the interfacial stress arising from the lattice mismatch between rhombohedral α-Fe_2_O_3_ (a ≈ 5.035 Å, c ≈ 13.75 Å) [29] and tetragonal anatase (a ≈ 3.784 Å, c ≈ 9.514 Å) [30]. At the Fe–O–Ti interfacial regions, shared oxygen atoms mediate bonding between phases, generating localized strain that increases with TiO_2_ loading. This explains the concurrent increase in dislocation density and microstrain for both hematite and anatase, particularly at HT33%, where the interfacial area is maximized.

Crystallinity followed a non-linear trend: at HT20–25%, the total crystalline fraction peaked (~98%), likely due to cooperative nucleation, whereas at HT33%, it decreased (~92%), reflecting the higher density of strained interfacial regions. At HT50%, hematite partially recovered in size (~21.5 nm) with reduced strain, while anatase stabilized the overall system, yielding ~94% crystallinity. These results demonstrate that the microstructural evolution of Fe_2_O_3_–TiO_2_ is governed not by solid-state substitution, but by interfacial lattice mismatch. This balance between ordered domains and strain-induced defects was particularly favorable in HT33%, where the density of Fe_2_O_3_–TiO_2_ interfaces was maximized, correlating with its superior photocatalytic performance; this is discussed in later sections of this paper.

### 2.2. Morphological and Surface Analysis and Elemental Distribution

**Figure 2:**
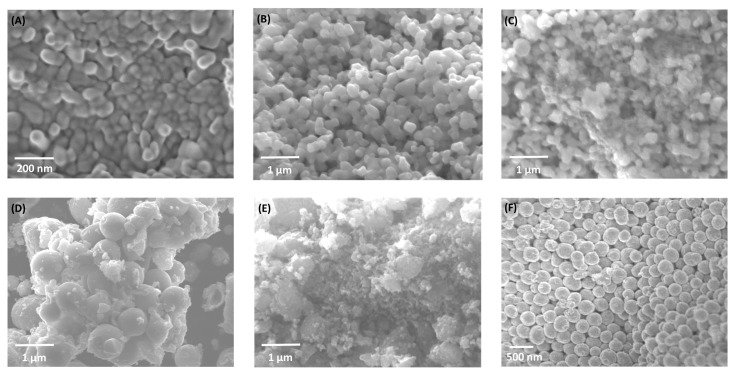
SEM micrographs of (**A**) H700 (α-Fe_2_O_3_), (**B**) HT1%, (**C**) HT20%, (**D**) HT33%, (**E**) HT50%, and (**F**) TiO_2_ samples synthesized using the solvothermal method. The series shows progressive TiO_2_ coating on hematite as the TiO_2_ content increases, leading to smoother and more compact aggregates.

Scanning electron microscopy (SEM) and energy-dispersive spectroscopy (EDS) were used to investigate morphological evolution in Fe_2_O_3_–TiO_2_ composites (Figure 2). Pure α-Fe_2_O_3_ (H700) displays irregular granular particles, whereas pure TiO_2_ forms uniform spherical nanostructures. Intermediate compositions (HT1–HT50%) reveal partial TiO_2_ coating over hematite grains, producing smoother surfaces and progressively more compact aggregates. These morphological changes suggest closer contact between Fe_2_O_3_ and TiO_2_ particles as the TiO_2_ fraction increases.

**Figure 3:**
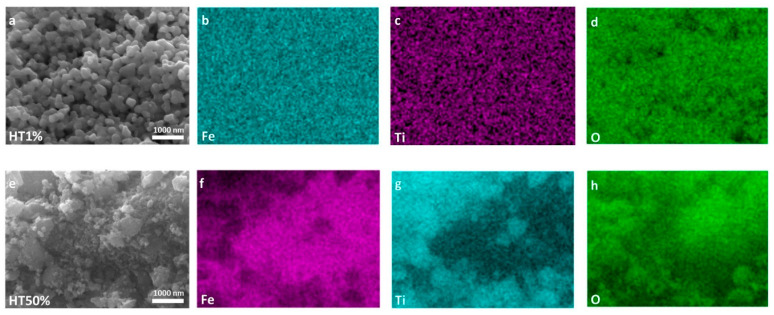
High-resolution SEM micrographs and EDS elemental maps of Fe, Ti, and O for Fe_2_O_3_–TiO_2_ composites. Panels (**a**–**d**) correspond to sample HT1%, while panels (**e**–**h**) correspond to HT50%. SEM images are shown in (**a**,**e**). Elemental distribution maps are shown for Fe in (**b**,**f**); for Ti in (**c**,**g**); and for O in (**d**,**h**).

To further explore the morphological and compositional evolution, SEM and EDS analyses were performed for two representative samples, HT1% and HT50% (the lowest and highest TiO_2_ contents, respectively), as shown in Figure 3. Panels (a–d) correspond to sample HT1%, and panels (e–h) to HT50%.

In the pure hematite sample, particles consisted of irregular aggregates formed of nanocrystallites with an average size of 54.7 ± 4 nm (Figure 2 and Figure S1). The surface exhibited a heterogeneous texture with faceted edges and distinct grain boundaries, typical of hematite obtained at elevated calcination temperatures. Moderate agglomeration was observed, with interparticle contact occurring primarily through grain coalescence, potentially limiting the accessible surface area.

Upon incorporation of 1% TiO_2_ (HT1%), the initial irregular morphology of hematite evolved into well-defined, semi-spherical particles with a highly homogeneous size distribution of approximately 195 ± 2.9 nm (Figure 2, Figure 3 and Figure S2). This pronounced increase in apparent particle size can be attributed to the conformal deposition of a thin TiO_2_ shell over the hematite cores, effectively smoothing surface asperities while preserving the overall particle integrity [17]. Elemental mapping using EDS confirmed the uniform spatial distribution of Fe, Ti, and O despite the minimal Ti content, and quantitative point analysis yielded 65.4% Fe, 34.4% O, and 0.2% Ti (Table 2 and Figure S3), in agreement with the nominal loading. The morphological uniformity observed in HT1% suggests reduced defect density on the outer surface, which may contribute to improved structural coherence and photocatalytic stability.

A markedly different microstructural scenario was observed for HT50% (Figure 2 and Figure 3). The semi-spherical trend was largely replaced by irregular and partially amorphous domains, accompanied by extensive agglomeration and the emergence of high-contrast regions attributable to TiO_2_-rich clusters. These features point to the formation of thick and discontinuous TiO_2_ coatings, with local coalescence of anatase grains, resulting in an overall increase in surface heterogeneity. EDS analysis indicated a composition of 44.2% Fe, 35.3% O, and 20.2% Ti (Table 2 and Figure S4), consistent with the intended synthesis ratio. The more complex surface topology of HT50% may introduce additional interfacial boundaries while reducing homogeneous active-site exposure, possibly limiting its photocatalytic performance [31].

The titanium content progressively increased, following the expected compositional trend. Slight variations inherent to semi-quantitative EDS measurements were observed; however, overall consistency with theoretical synthesis ratios was observed.

This morphological and compositional analyses complement our XRD findings, confirming effective incorporation and homogeneous distribution of TiO_2_ within the hematite matrix. These structural modifications could influence the composites’ photocatalytic and adsorption properties, as discussed in subsequent sections.

The surface area and porosity of the samples was studied using N_2_ adsorption–desorption analysis. Considering the extensive set of compositions, a representative subset was selected for BET analysis—H700 (pure Fe_2_O_3_), TiO_2_, and HT33%—chosen to capture both extremes and the optimal ratio within the series.

**Figure 4:**
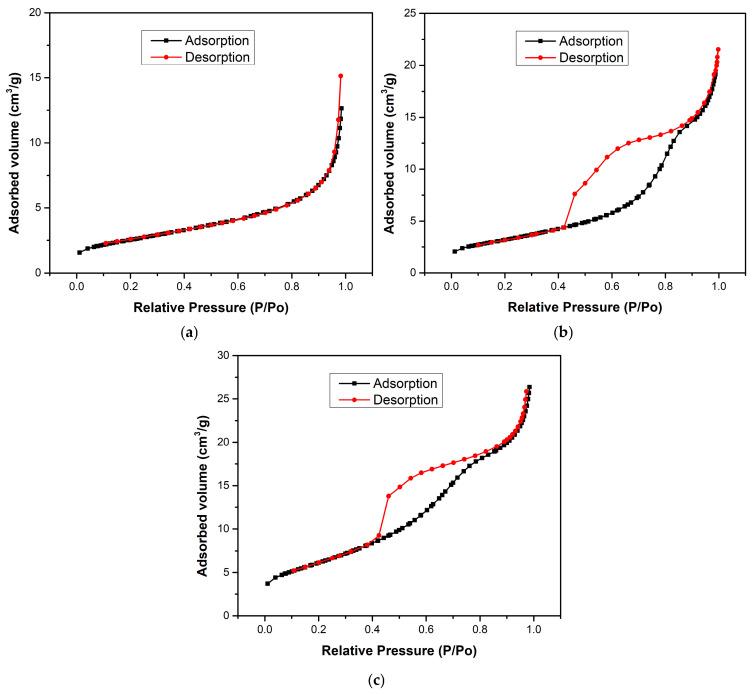
The BET isotherms of (**a**) H700, (**b**) TiO_2_, and (**c**) HT33% samples.

Figure 4 shows the BET isotherms of H700 (pure Fe_2_O_3_), TiO_2_, and HT33% composites. H700 exhibits a type-III isotherm without hysteresis, consistent with weak adsorbate–adsorbent interactions and the dominance of non-accessible/macroporous voids formed by interparticle contacts [32]. In contrast, TiO_2_ and HT33% display type-IV isotherms with pronounced hysteresis, characteristic of mesoporous networks. The hysteresis shape (a steep desorption “knee” around P/P_0_ ≈ 0.45–0.55 and a delayed closure) corresponds to an H2-type loop, which is typically associated with pore blocking and/or constricted-neck (“ink-bottle”) mesopores within disordered aggregates rather than uniform cylindrical pores [33]. This classification contrasts with the type-III isotherm without hysteresis observed for H700, confirming its low adsorptive affinity and macroporous character.

Quantitatively, the specific surface area increases from 9.1 m^2^·g^−1^ (H700) to 11.52 m^2^·g^−1^ (TiO_2_) and reaches 22.47 m^2^·g^−1^ for HT33%, i.e., a ~2.5× gain versus pure hematite (Table 3). The total pore volume almost doubles in HT33% (0.0435 vs. 0.0221 cm^3^·g^−1^), while the average pore diameter decreases from 15.2 nm (H700) to 10.7 nm (TiO_2_) and 8.1 nm (HT33%).

SEM–EDS micrographs (Figure 2 and Figure 3) confirm this morphological correlation: TiO_2_ nanoparticles are distributed over the Fe_2_O_3_ surface rather than forming a core–shell architecture, producing clusters and contact regions where interparticle voids and necked mesopores develop. These morphological features are consistent with H2-type hysteresis, supporting that the mesoporosity arises from the disordered stacking of both oxides and the partial coverage of hematite grains by TiO_2_. The apparent observed porosity and particle coalescence trends across all compositions (seen previously in SEM) are qualitatively consistent with the expected variation in specific surface area, supporting the overall interpretation of a progressive textural evolution along the series

From a photocatalytic perspective, the increase in specific surface area and mesopore fraction increases the population of accessible surface sites exposed to light and facilitates the diffusion and adsorption of methylene blue molecules within the catalyst network [34]. Thus, the improved texture of HT33% provides a structural basis for its superior photocatalytic activity compared to its single-oxide counterparts.

### 2.3. Spectroscopic Analysis: Diffuse Reflectance Spectroscopy and Fourier-Transform Infrared Spectroscopy

**Figure 5:**
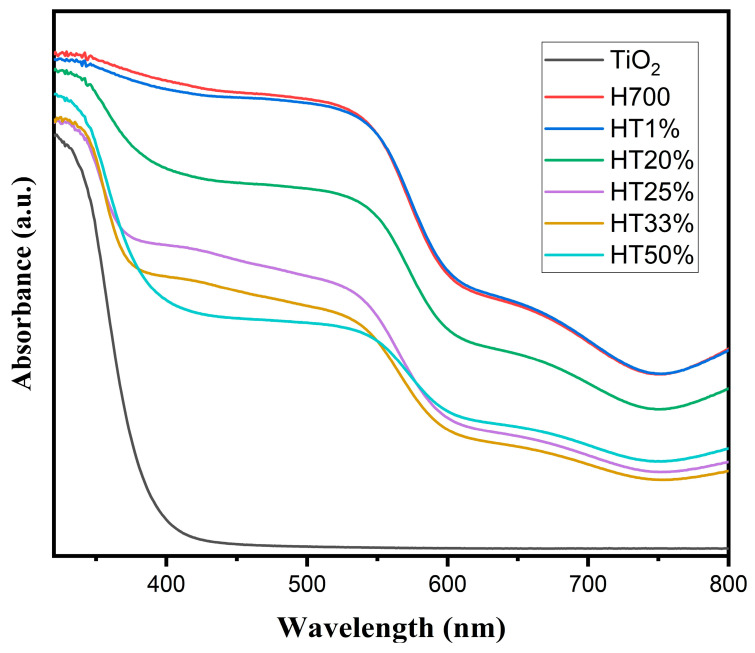
UV–Vis diffuse reflectance spectra of H700 (pure hematite), TiO_2_, and Fe_2_O_3_–TiO_2_ composites with increasing TiO_2_ content (HT1% to HT50%).

Diffuse reflectance spectroscopy (DRS) was employed to investigate the optical behavior of the binary composites and to evaluate the influence of increasing TiO_2_ content on light absorption. Figure 5 displays the DRS spectra of pure hematite (H700), pure TiO_2_, and the Fe_2_O_3_–TiO_2_ composites.

The absorption spectrum of pure TiO_2_ exhibited a sharp edge at approximately 395 nm, characteristic of anatase-phase TiO_2_, indicating strong absorption in the UV region and minimal absorption in the visible range.

In contrast, the H700 (pure hematite) spectrum showed a broad absorption band extending throughout the visible region, with significant absorbance between 650 and 400 nm, consistent with its narrow band gap (~2.0 eV) and well-known visible-light responsiveness.

The HT1% composite displayed a profile nearly identical to that of H700, suggesting that the small amount of TiO_2_ had a negligible optical influence. As TiO_2_ content increased (HT20% to HT50%), a gradual reduction in visible absorption intensity was observed, particularly in the 500–650 nm range. This trend reflects a decreasing contribution from hematite and the increasing dominance of the TiO_2_ optical response within the composite.

As TiO_2_ content increased across the binary composites, a progressive decrease in the visible-light absorption shoulder was observed, particularly in the range of 500–650 nm, which corresponds to the optical contribution of hematite. This attenuation suggests the diminishing optical influence of α-Fe_2_O_3_ as the anatase phase becomes more dominant. In HT33% and HT50%, the absorption features more closely resemble those of pure TiO_2_, indicating that the optical behavior is increasingly governed by the TiO_2_ phase, rather than by hematite. Notably, no significant shift in the absorption edge’s position was observed, but rather, a modulation in the relative intensities of absorption bands associated with each component was observed [35].

These changes in optical response correlate with the compositional evolution of the samples, as confirmed using EDS, which showed a progressive decrease in iron content and a corresponding increase in titanium content with increasing TiO_2_ proportions. The reduction in visible-light absorption, particularly in the 500–650 nm range, can therefore be attributed to a lower hematite contribution per unit mass. This compositional shift alters the optical balance within the composites, leading to the increasing dominance of the anatase TiO_2_ phase in the response. Although structural defects such as microstrain and dislocation density were found to increase with TiO_2_ content, their contribution to overall optical absorption is likely marginal.

Importantly, this tunable optical behavior demonstrates the potential of Fe_2_O_3_–TiO_2_ composites to be tailored for photocatalytic applications under specific light regions.

Band gap energies (Eg) were estimated from Tauc plots constructed using the absorbance spectra from diffuse reflectance spectroscopy. The term (A⋅hν)^1/2^ was plotted against photon energy (hν), assuming indirect allowed transitions (m = 2) for anatase TiO_2_ and α-Fe_2_O_3_. Eg was determined by extrapolating the linear region of the absorption edge to a defined baseline [36] (Figure S5), rather than to the X-axis, in order to avoid artificial shifts in the calculated band gap [18,37]. The resulting values are summarized in Table 4.

As shown in Table 4, pure hematite (H700) exhibited a band gap of 1.99 ± 0.02 eV, consistent with the reported values for α-Fe_2_O_3_ (1.9–2.1 eV) [38]. Pure anatase TiO_2_ exhibited a band gap of 3.21 ± 0.04 eV, in agreement with typical values for nanoscale anatase (3.2–3.3 eV) [35]. For the HT1% composite, only an Fe_2_O_3_ transition was detected (2.06 ± 0.02 eV), while no clear TiO_2_ edge was observed, most likely due to its very low concentration, which prevented its optical signature from being resolved. In the composites with higher TiO_2_ content, the Fe_2_O_3_ band gap remained nearly constant (2.06–2.08 eV), suggesting that the fundamental electronic transition of hematite was preserved upon incorporating TiO_2_ [11]. In contrast, the TiO_2_ band gap appeared at HT20% (3.25 ± 0.06 eV) and progressively shifted toward higher energies with increasing TiO_2_ content, reaching 3.35 ± 0.09 eV at HT50%. This slight blue shift can be attributed to nanoscale effects and interfacial strain [19], as also evidenced by the microstructural analysis. The coexistence of two distinct absorption edges in the composites confirms the formation of Fe_2_O_3_–TiO_2_ heterostructures [39], which modulate light harvesting across both the visible and UV regions. Importantly, this dual optical response underpins the enhanced photocatalytic performance observed in intermediate compositions (HT20–HT33%), where an optimal balance between visible-light absorption (hematite) and UV absorption by TiO_2_ is achieved.

Fourier-transform infrared (FTIR) spectroscopy was employed to investigate chemical interactions between hematite and TiO_2_ phases and to track changes in characteristic absorption bands with varying TiO_2_ content (Figure S6). In the HT1% composite, two distinct bands at 543 cm^−1^ and 464 cm^−1^ were clearly observed, corresponding to Fe–O stretching vibrations characteristic of hematite [24]. For intermediate- and high-TiO_2_ compositions (HT20% to HT50%), these hematite-related bands became progressively broader and less intense, likely due to spectral overlap with Ti–O–Ti stretching and bending vibrations. Anatase-phase TiO_2_ typically exhibits a broad absorption feature in the 400–750 cm^−1^ region, with maxima commonly centered around ~500–600 cm^−1^. The superposition of both oxide bands complicates individual assignment at higher TiO_2_ loadings.

### 2.4. Photocatalytic Activity

**Figure 6:**
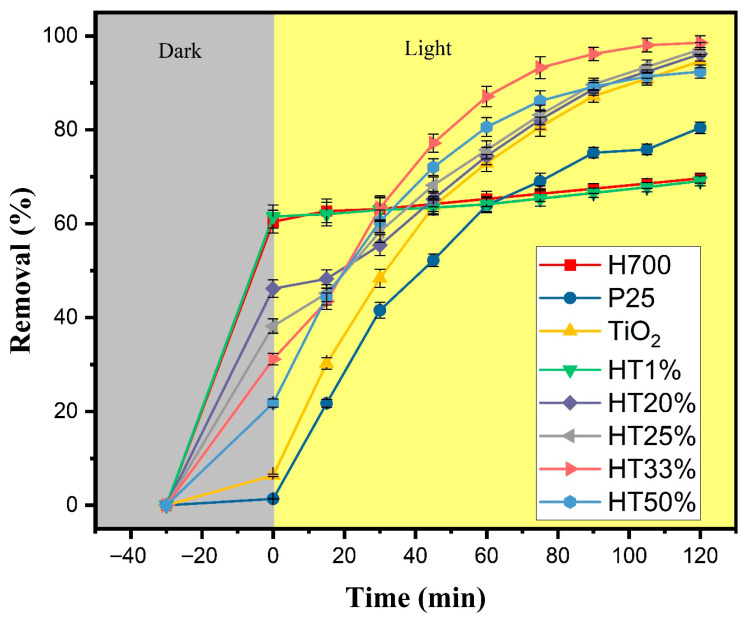
Total removal of methylene blue (10 µM) as a function of time for Fe_2_O_3_–TiO_2_ composites, pure TiO_2_, P25, and H700. The gray region represents the adsorption phase in darkness (30 min), whereas the yellow region corresponds to the photocatalytic phase under xenon light irradiation (120 min). Error bars represent the standard deviation of two independent experiments performed under identical conditions (*n* = 2).

Figure 6 displays the methylene blue (MB) removal profiles over time for the Fe_2_O_3_–TiO_2_ binary composites with different TiO_2_ proportions under the following two-phase test: 30 min of adsorption in the dark, followed by 120 min of photocatalysis under xenon lamp irradiation. Possible direct photolysis of MB was evaluated without any catalyst under identical irradiation conditions. The absorbance maximum decreased by only 6%, indicating minor self-photolysis (Figure S7).

During the dark adsorption phase, a clear inverse correlation between TiO_2_ content and adsorption capacity was observed. Samples with lower TiO_2_ content, such as HT1%, exhibited high initial removal (~62%), likely due to the dominant presence of hematite, which is known for its strong affinity toward methylene blue. Previous studies have demonstrated (by DFT calculations) that nitrogen atom in MB forms hydrogen bonds with surface hydroxyl groups on hematite [40]. In contrast, HT33% and HT50% showed lower adsorption (~49% and ~42%, respectively), consistent with the reduced contribution of hematite and the relatively poor adsorption capacity of TiO_2_ [41].

Under light irradiation, all binary composites demonstrated improved dye degradation in comparison with hematite and anatase, with HT33% showing the best performance, achieving ~98% total removal. This indicates its superior photocatalytic efficiency and balanced performance in terms of both adsorption and photoreactivity [42]. The HT25% and HT20% samples also showed removal efficiencies above 90%, suggesting the existence of synergistic effects between TiO_2_ and Fe_2_O_3_ even when TiO_2_ content was moderate [20]. In contrast, HT1% performed similarly to pure hematite, indicating limited photocatalytic enhancement when TiO_2_ content is very low.

These trends align with those observed in prior structural and optical analyses. The HT20% to HT33% samples represent an optimal compromise between surface adsorption capacity and photocatalytic activity. The presence of hematite contributes primarily to dye adsorption, as evidenced by the strong removal efficiency in the dark phase, whereas TiO_2_ provides the photoactive sites responsible for light-induced reactions, leading to dye degradation. In this sense, the enhanced performance of the intermediate composites could possibly arise from a complementary effect: hematite promotes dye–surface interactions—possibly through a capacitive double-layer contribution—while TiO_2_ enhances the utilization of photogenerated charge carriers at the catalyst surface. Additionally, the favorable microstructural features observed in this range—such as high crystallinity and moderate crystallite size—may improve charge utilization and reduce recombination likelihood, contributing to overall efficiency. Conversely, HT50%, despite its higher TiO_2_ content, shows a slight drop in performance, likely due to reduced dye interaction caused by lower hematite content.

Taken together, these data indicate that ~33% TiO_2_ is the optimal proportion for maximizing both adsorption and photocatalytic performance; this balance is further explored below in our kinetic analysis.

**Figure 7:**
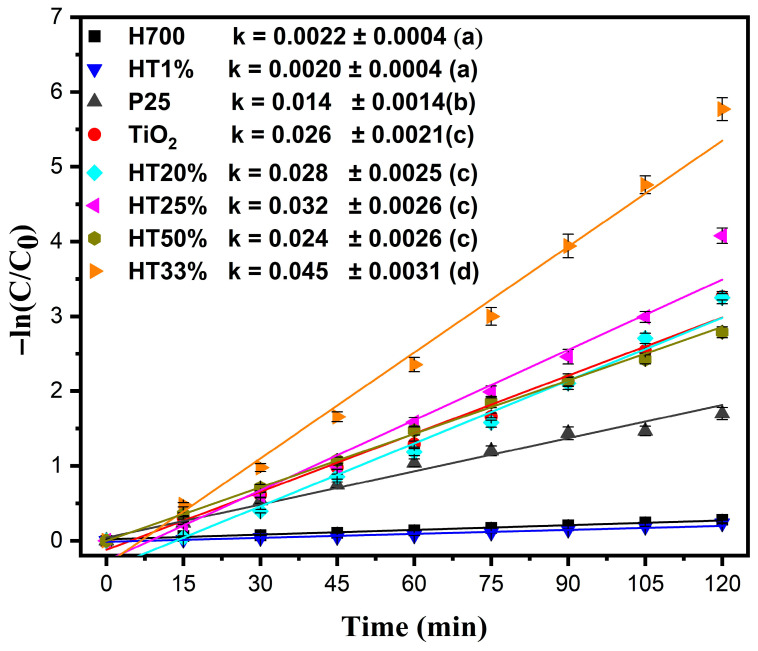
Pseudo-first-order kinetic plots for the photocatalytic degradation of methylene blue (10 µM) by Fe_2_O_3_–TiO_2_ composites, pure TiO_2_, P25, and H700. The data were fitted using the equation −ln(C/C_0_) = kt. Error bars represent the standard deviation of two independent experiments performed under identical conditions (*n* = 2). Comparative values of the rate constants (k min^−1^) for all samples are shown in the legend; Tukey groups are shown in parentheses.

Figure 7 shows the pseudo-first-order kinetic plots, following the equation $-\ln\left(\frac{C}{C_{0}}\right)=kt$, where C denotes the concentration of methylene blue at time t (mg/L), C_0_ denotes the initial concentration of methylene blue (mg/L), t denotes the reaction time (min), and k denotes the apparent rate constant of the pseudo-first-order reaction (min^−1^) [43] (non-linear pseudo-first-order fitting is presented in Figure S8).

The highest kinetic constant was observed for HT33% (k = 0.045 min^−1^). Since the rate constant directly reflects the degradation rate in the pseudo-first-order model, a higher k value is indicative of faster reaction kinetics and therefore superior photocatalytic activity. The next-highest kinetic constants were observed for HT25% and HT20%, with k = 0.032 min^−1^ and k = 0.028 min^−1^, respectively—surpassing even pure TiO_2_ (k = 0.026 min^−1^) and demonstrating the synergistic behavior of the binary composites [19]. Despite containing more TiO_2_, HT50% exhibited a slightly lower reaction rate (k = 0.024 min^−1^), likely due to dye–surface interactions reducing as the hematite content decreased, limiting adsorption and interfacial contact.

The kinetic behavior of HT1% (k = 0.0020 min^−1^) was nearly identical to that of pure hematite (H700, k = 0.0022 min^−1^), confirming that very low TiO_2_ content does not significantly enhance photocatalytic performance. Additionally, the commercial P25 TiO_2_ showed a lower reaction rate (k = 0.014 min^−1^) than the solvothermally synthesized TiO_2_ used in this study, underscoring the superior structural and functional quality of the prepared material. Statistical analysis using a one-way ANOVA followed by Tukey’s HSD test (α = 0.05) confirmed that the differences among the rate constants were significant, (*p* < 0.0001). HT33% exhibited the highest k value, whereas H700 and HT1% were statistically similar and the least active (see the legends in Figure 7).

A higher surface area may lead to a higher adsorption capacity and higher photoactivity. However, the H700 sample with the lowest surface area had the best adsorption capacity. The surface area-normalized rate constant (k_s_ = k/surface area) was calculated for the H700, TiO_2_, and HT33% samples to consider the effect of the specific surface area [44]. The obtained k_s_ values were 0.0002, 0.0023, and 0.0020 L/(m^2^ min), respectively. TiO_2_ and HT33% showed nearly identical k_s_ values, confirming the role of surface area and mesoporous structure in the nanocomposite photocatalytic process [45]. While BET data were obtained only for representative samples, SEM observations revealed consistent morphological trends suggesting a similar textural evolution across the series.

To verify visible-light activity, additional photocatalytic tests were carried out using a UV-free Verasol LED lamp (100 mW cm^−2^). Under visible-only illumination, the HT33% composite exhibited a higher apparent rate constant than bare TiO_2_ (k = 0.009 min^−1^ vs. 0.004 min^−1^), confirming its ability to operate under visible light. As expected, all rates were substantially lower than those obtained under xenon irradiation due to the absence of UV photons (see Figure S9).

Overall, these kinetic results point to a multifactorial mechanism: the enhanced performance of HT20–HT33% arises from a combination of the effective dye adsorption enabled by hematite and the efficient light-driven processes associated with TiO_2_ domains. These interfacial effects are further supported by favorable microstructural characteristics such as a moderate crystallite size, high crystallinity, and a balanced distribution of structural defects, which may promote more efficient charge utilization at the catalyst surface. Rather than being governed by a single variable, the observed activity reflects a delicate balance between surface accessibility and photoinduced processes operating at the semiconductor interface.

### 2.5. Reusability and Stability

**Figure 8:**
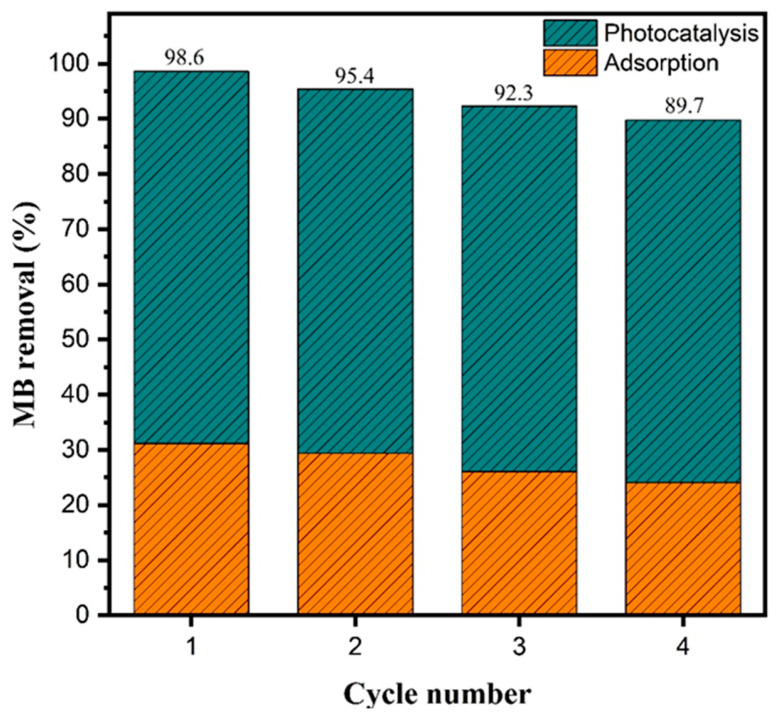
Photocatalytic reusability of HT33% under xenon lamp irradiation (AM 1.5 G, 100 mW cm^−2^) over four consecutive cycles.

The reusability of a photocatalyst is a key metric of its practical applicability and long-term stability in wastewater treatment processes [46]. To assess this property, the Fe_2_O_3_–TiO_2_ composite with 33% TiO_2_ (HT33%) was tested over four consecutive photocatalytic cycles under identical experimental conditions (Figure 8).

The total methylene blue removal decreased from 98.6% in the first cycle to 89.7% in the fourth, corresponding to an overall reduction of approximately 9% after four uses. The relative contributions of adsorption and photocatalysis remained nearly constant, with adsorption decreasing from 31.1% to 24.1% and the photocatalytic contribution varying slightly from 67.4% to 65.7%. These results indicate that the material retained consistent photocatalytic behavior over successive runs, with only minor quantitative variations, confirming its potential for practical applications in water purification systems.

FTIR spectra of HT33% composite before and after the reusability cycles, showing the characteristic Fe–O stretching bands at 543 and 464 cm^−1^ (Figure S10). The spectra remain very similar, indicating high structural stability after repeated photocatalytic use.

### 2.6. Energy-Band Correlation and Possible Charge-Transfer Mechanism

**Figure 9:**
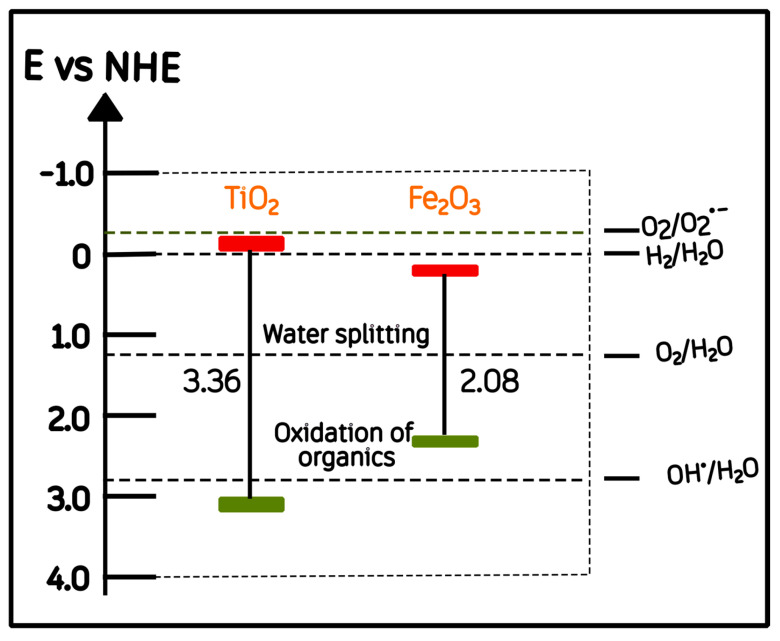
Schematic energy-band correlation of α-Fe_2_O_3_ and anatase TiO_2_ relative to the normal hydrogen electrode (NHE). Red and green bars indicate the conduction and valence band energy levels, respectively.

Our optical results (Section 2.3) revealed two distinct absorption edges corresponding to α-Fe_2_O_3_ (Eg ≈ 2.07 eV) and anatase TiO_2_ (Eg ≈ 3.30 eV). Based on these experimental values and the conduction-band (CB) potentials reported in the literature for TiO_2_ and Fe_2_O_3_ (−0.22 and 0.3 V vs. NHE, respectively) [47,48,49], an energy-band correlation diagram prior to junction formation was constructed (Figure 9).

**Figure 10:**
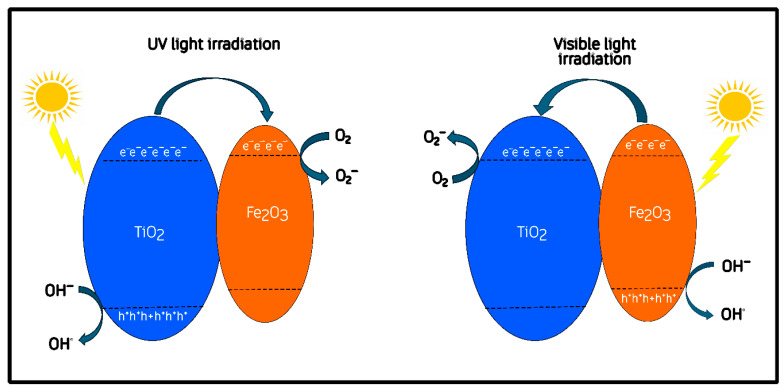
Proposed charge-transfer mechanism for the Fe_2_O_3_/TiO_2_ heterojunction under UV and visible-light irradiation.

It has been proposed that once Fe_2_O_3_ and TiO_2_ are brought into close contact, their differing functions can drive Fermi-level equilibration, which induces interfacial band bending and the formation of a built-in electric field at the junction [50,51]. These effects, commonly re-ported for semiconductor heterojunctions, are generally regarded as important factors for promoting directional charge separation and interfacial carrier transport [52,53]. After the heterojunction is formed, several authors have further suggested that the conduction band of α-Fe_2_O_3_ is located slightly above (i.e., more negative in energy) that of TiO_2_ once equilibrium is established [49,54]. This rearrangement has been proposed to facilitate the migration of photoexcited electrons from Fe_2_O_3_ toward TiO_2_ under visible light, while holes remain in the hematite phase [52]. This post-contact configuration is represented conceptually in Figure 10.

Although this configuration has been widely proposed for Fe_2_O_3_/TiO_2_ heterostructures, it should be noted that no direct experimental evidence (such as PL quenching or photocurrent response) was obtained in the present study. Therefore, this diagram and the subsequent discussion should be regarded as a plausible but unverified description of the processes that could occur in our system and which still require experimental confirmation.

The charge-transfer mechanism following junction formation is depicted in Figure 10. In this configuration, the equilibration of Fermi levels and the resulting band bending are expected to promote charge separation across the Fe–O–Ti interface [51]. Under visible-light excitation, α-Fe_2_O_3_ acts as the primary absorber, generating electron–hole pairs whose separation may be assisted by the interfacial field: electrons can migrate toward TiO_2_, while holes remain within hematite [49,50]. Under UV illumination, TiO_2_ dominates photon absorption and generates additional carriers that participate in oxidation and reduction pathways near the interface [55,56]. According to previous reports, electrons accumulated on TiO_2_ can participate in the reduction in adsorbed O_2_ to superoxide radicals (O_2_^·−^), whereas holes on Fe_2_O_3_ may contribute to the oxidation of methylene-blue molecules or surface hydroxyls to produce ·OH radicals [57,58].

This interfacial synergy, supported by the structural coherence and moderate defect density observed for the intermediate composition (HT33%), provides a consistent qualitative explanation for its superior photocatalytic activity.

The mechanism proposed here is consistent with the general heterojunction models in which an interfacial field is considered to assist charge separation, aligning with the type-II or S-scheme configurations reported for Fe_2_O_3_/TiO_2_ composites in the recent literature [59,60]. Figure 9 and Figure 10 thus represent a literature-supported qualitative interpretation linking the optical band gaps and structural observations to the photocatalytic behavior obtained in this work.

## 3. Materials and Methods

### 3.1. Materials

All chemicals were used as received without further purification. Iron (III) chloride hexahydrate (FeCl_3_·6H_2_O, ≥97%), ammonium hydroxide solution (NH_4_OH, 28–30%), titanium (IV) isopropoxide (97%), triethylamine (≥99.5%), and absolute ethanol (≥99.5%) were purchased from Sigma-Aldrich Chemical Co. (St. Louis, MO, USA). Deionized water (18.2 MΩ·cm) was used in all preparations, and commercial TiO_2_ (P25, Sigma-Aldrich) was employed as a reference photocatalyst for comparison. Methylene blue (MB, C_16_H_18_ClN_3_S, ≥95%, Sigma-Aldrich) was used as a model organic dye pollutant for photocatalytic degradation experiments.

### 3.2. Synthesis of Hematite Nanoparticles (α-Fe_2_O_3_)

Hematite nanoparticles (α-Fe_2_O_3_) were synthesized via a co-precipitation method. In brief, 4.054 g of iron (III) chloride (FeCl_3_·6H_2_O) was dissolved in 150 mL of deionized water. The obtained solution was filtered through a 0.25 µm membrane filter and then heated to 80 °C under vigorous stirring for 1 h. Subsequently, the solution was slowly titrated with concentrated ammonium hydroxide (NH_4_OH, 28–30%) until a final pH of 11 was reached. The formed precipitate was stirred for an additional 4 h, centrifuged at 4500 rpm for 10 min, and repeatedly washed with deionized water to remove residual ions and impurities. Then, the clean precipitate was dried at 80 °C for 24 h and calcined at 700 °C for 4 h to obtain crystalline hematite nanoparticles (designated as H700), which exhibited optimal photocatalytic performance according to our previous tests.

### 3.3. Synthesis of TiO_2_ Nanoparticles and Fe_2_O_3_-TiO_2_ Binary Composites

TiO_2_ nanoparticles were synthesized using a solvothermal method. Briefly, 480 µL of titanium (IV) isopropoxide (97%) and 400 µL of triethylamine (≥99.5%) were added dropwise into 40 mL of absolute ethanol under vigorous stirring. The resulting homogeneous solution was transferred into a 100 mL Teflon-lined stainless steel autoclave and heated at 200 °C for 20 h. After cooling to room temperature, the obtained white precipitate was centrifuged at 6000 rpm for 20 min and washed three times with absolute ethanol and deionized water to remove unreacted precursors and organic residues. The solid was dried at 60 °C for 12 h and subsequently calcined at 450 °C for 2 h to obtain crystalline TiO_2_.

Fe_2_O_3_–TiO_2_ binary composites were synthesized with a similar solvothermal approach, using the previously synthesized hematite nanoparticles (H700). Briefly, 80 mg of H700 nanoparticles were dispersed in 80 mL of absolute anhydrous ethanol using ultrasonication for 1 h to ensure homogeneous dispersion. Subsequently, 400 µL of triethylamine and calculated amounts of titanium (IV) isopropoxide were added to achieve binary composites with the following molar proportions of TiO_2_ relative to hematite: 1%, 20%, 25%, 33.3%, and 50%. The resulting mixtures were stirred for 1 h, transferred into a 100 mL Teflon-lined stainless steel autoclave, and subjected to solvothermal treatment at 200 °C for 20 h. After the solvothermal reaction, the obtained composites were centrifuged at 6000 rpm for 20 min and washed three times with absolute ethanol and distilled water to remove impurities and organic residues. Finally, the samples were dried at 60 °C for 12 h and calcined at 450 °C for 2 h.

The synthesized composites are hereafter denoted as HT1%, HT20%, HT25%, HT33%, and HT50%, corresponding to hematite–TiO_2_ molar ratios of 99:1, 80:20, 75:25, 66.6:33.3, and 50:50, respectively.

### 3.4. Photodegradation of Methylene Blue

Photodegradation is one of the most widely used methodologies for evaluating the dye removal efficiency of photocatalytic materials. In this study, methylene blue (MB) aqueous solutions with a concentration of 10 μM were prepared for each composite material, using a total volume of 80 mL. A catalyst loading of 1.0 g·L^−1^ (corresponding to 80 mg of photocatalyst in 80 mL of solution) was used in all experiments. Prior to illumination, the suspensions were kept in the dark for 30 min under constant stirring to evaluate the contribution of adsorption processes.

Subsequently, the samples were irradiated using a xenon arc lamp equipped with an AM 1.5 global filter, calibrated to deliver an irradiance of 100 mW/cm^2^ by adjusting the lamp-to-sample distance. The photocatalytic tests were conducted for 120 min, with 1.5 mL aliquots collected every 15 min. Each aliquot was immediately centrifuged, and the supernatant was analyzed using UV–Vis spectrophotometry, with the decrease in absorbance monitored at 664 nm, which corresponds to the maximum absorption wavelength of methylene blue [43].

All experiments were performed in duplicate, and the average values with standard deviations (±SD) are reported. Apparent rate constants (k) were determined by fitting the photocatalytic data to the pseudo-first-order model (−ln(C/C_0_) = kt) after the 30 min dark adsorption stage, and the quality of the fits was evaluated using the corresponding correlation coefficients (R^2^).

The reusability of the Fe_2_O_3_–TiO_2_ composites was evaluated through four consecutive photocatalytic cycles under identical experimental conditions. Each run consisted of 30 min of dark adsorption followed by 120 min of illumination using the xenon arc lamp. The catalyst loading was fixed at 1.0 g·L^−1^ in all tests.

After each cycle, the suspension was centrifuged at 6000 rpm for 10 min to recover the photocatalyst. The solid was washed with deionized water and absolute ethanol and then dried at 60 °C for 12 h before reuse. The subsequent photocatalytic runs were conducted under the same conditions. The photocatalytic performance in each cycle was determined using UV–Vis spectrophotometry at 664 nm, based on the total methylene blue removal after 120 min of illumination.

### 3.5. Characterization Techniques

The structural, morphological, chemical, and optical properties of the synthesized materials were characterized using multiple techniques. The X-ray diffraction (XRD) patterns of α-Fe_2_O_3_, TiO_2_, and the Fe_2_O_3_–TiO_2_ binary composites were collected using a Bruker D2 Phaser diffractometer with Bragg–Brentano geometry (Bruker Corporation, Billerica, MA, USA), equipped with a Cu Kα radiation source (λ = 1.5418 Å), in the 2θ range of 20–80°. The surface morphology and elemental composition were analyzed using a field-emission scanning electron microscope (FE-SEM, JEOL JSM-7800F, Tokyo, Japan) coupled with energy-dispersive X-ray spectroscopy (EDS, X-Max 80, Oxford Instruments, Abingdon, UK).

Fourier-transform infrared (FTIR) spectra were recorded on a PerkinElmer Frontier spectrometer (Waltham, MA, USA) in the 4000–400 cm^−1^ range using an attenuated total reflectance accessory. UV–Vis diffuse reflectance spectra (DRS) of all powdered materials were acquired with a UV–Vis–NIR spectrophotometer (Cary 5000i, Agilent Technologies, Santa Clara, CA, USA) equipped with an integrating sphere, operating in the 300–800 nm range, with BaSO_4_ as the reference standard. Textural properties of the samples were determined using N_2_ adsorption–desorption measurements using a Micromeritics, TriStar II Plus analyzer (Norcross, GA, USA).

## 4. Conclusions

This study demonstrates that the photocatalytic performance of Fe_2_O_3_–TiO_2_ composites strongly depends on the TiO_2_ loading and the resulting interfacial interactions. The structural analyses revealed that increasing TiO_2_ content reduces crystallite size and induces lattice distortions, while the optical studies confirmed that band gap modulation arises from the coexistence of hematite and anatase phases. The morphological observations showed that moderate TiO_2_ incorporation preserves structural homogeneity, whereas excessive loading promotes agglomeration and reduces photocatalytic efficiency. The photocatalytic tests evidenced that the composite with 33% TiO_2_ achieved the optimal balance between crystallinity, defects, and band gap tuning, reaching 98% methylene blue removal and a superior rate constant compared to both pristine Fe_2_O_3_ and commercial TiO_2_. These findings highlight that tailoring TiO_2_ content is a simple yet effective strategy for designing cost-efficient Fe_2_O_3_–TiO_2_ heterostructures suitable for water purification.

## Figures and Tables

**Table 1:** Microstructural parameters of Fe_2_O_3_–TiO_2_ composites, including crystallite size, dislocation density, microstrain, and crystallinity.

Sample	Phase	D (nm)	δ/×10^14^ (m^−2^)	ε/×10^−3^	Xc (%)	Total Crystallinity (%)
HT1%	Hematite (α-Fe_2_O_3_)	19.99	2.55	4.36	69.75	69.75
HT20%	Hematite (α-Fe_2_O_3_)	24.65	2.83	3.86	52.24	97.73
	TiO_2_ (anatase)	10.46	10.85	9.98	47.76	
HT25%	Hematite (α-Fe_2_O_3_)	21.55	4.12	5	49.98	98.56
	TiO_2_ (anatase)	10.73	10.91	9.7	50.02	
HT33%	Hematite (α-Fe_2_O_3_)	15.76	6.58	4.7	53.32	92.48
	TiO_2_ (anatase)	10.44	11.09	9.78	46.68	
HT50%	Hematite (α-Fe_2_O_3_)	21.47	3.74	4.58	50.91	94.38
	TiO_2_ (anatase)	9.4	13.39	10.96	49.09	

**Table 2:** EDS compositional analysis of Fe_2_O_3_–TiO_2_ binary composites.

Sample	Fe (%)	Ti (%)	O (%)
HT1%	65.4	0.2	34.4
HT20%	58.6	6.1	35.7
HT25%	55.4	7.7	36.5
HT33%	49.5	14.4	36.0
HT50%	44.2	20.2	35.3

**Table 3:** Textural properties of H700, TiO_2_, and HT33% samples.

Samples	Surface Area (m^2^/g)	Pore Size (nm)	Pore Volume (cm^3^/g)
H700	9.1	15.2	0.02207
TiO_2_	11.52	10.7	0.02959
HT33%	22.47	8.1	0.04351

**Table 4:** Band gap energies (Eg) and absorption edges for binary composites and reference materials.

Sample	Band Gap (eV) Fe_2_O_3_	Band Gap (eV) TiO_2_
H700	1.99 ± 0.02	-
TiO_2_	-	3.21 ± 0.04
HT1%	2.06 ± 0.02	-
HT20%	2.08 ± 0.01	3.25 ± 0.06
HT25%	2.08 ± 0.03	3.36 ± 0.08
HT33%	2.08 ± 0.02	3.35 ± 0.06
HT50%	2.07 ± 0.04	3.35 ± 0.09

## Data Availability

The original contributions presented in this study are included in the article/Supplementary Materials. Further inquiries can be directed to the corresponding author.

## Supplementary Materials

The following supporting information can be downloaded at: https://www.mdpi.com/article/10.3390/molecules30214309/s1. Figure S1: Particle size distribution of hematite nanoparticles (H700). Average size D = 54.7 ± 4 nm. Figure S2: Particle size distribution of HT1%. Average size D = 195 ± 2.9 nm. Figure S3: EDS spectrum of the HT1% sample showing the elemental composition. Figure S4: EDS spectrum of the HT50% sample showing the elemental composition. Figure S5: Tauc plots obtained from UV–Vis diffuse absorbance spectra of the Fe_2_O_3_–TiO_2_ binary composites: (a) HT1%, (b) HT20%, (c) HT25%, (d) HT33%, and (e) HT50%. The optical band gap values were estimated by extrapolating the linear fitting of the absorption edge to a defined baseline rather than to the photon energy axis, in order to avoid underestimation due to sub-band gap absorption. Figure S6: (a) FTIR spectra of Fe_2_O_3_–TiO_2_ composites with increasing TiO_2_ content and (b) the magnification of the 1000-400 cm^−1^ region. The main Fe–O stretching vibrations are visible around 543 and 464 cm^−1^. Figure S7: UV–Vis spectra of methylene blue (10 µM) before and after 120 min Xe irradiation (photolysis control). The absorbance decreased by ≈ 6% at λmax = 664 nm, indicating minor self-photolysis under AM 1.5 G equivalent illumination. Figure S8: Non-linear pseudo-first-order fitting of methylene blue photodegradation kinetics for Fe_2_O_3_–TiO_2_ composites. Experimental data (symbols) are well described by exponential decay models (solid lines), yielding reduced chi-square (χ^2^) values between 2.06 × 10^−4^ and 5.12 × 10^−3^, confirming the suitability of the model. Among all samples, HT33% showed the highest photocatalytic efficiency. Figure S9: (A) Pseudo-first-order kinetics (−ln(C/C_0_) vs. time) for TiO_2_ and HT33% composites under xenon (Xe) and UV-free Verasol LED (Ve) illumination, both adjusted to 1 Sun (100 mW cm^−2^). Apparent rate constants (k, min^−1^) were obtained from linear fits of the experimental data. Error bars represent the standard deviations from duplicate measurements. (B) Spectral irradiance distribution (ϕλ) of the xenon and Verasol sources. The xenon lamp covers the UV–visible–NIR region (300–1100 nm), whereas the Verasol LED emits exclusively in the visible range (λ > 400 nm). The lower photocatalytic rates observed under visible-only conditions confirm that HT33% retains measurable activity in the absence of UV photons. Figure S10: FTIR spectra of HT33% composite before and after the reusability cycles, showing the characteristic Fe–O stretching bands at 543 and 464 cm^−1^. The spectra remain very similar, indicating high structural stability after repeated photocatalytic use.
