# Supplementary material for: Photocatalytic Evaluation of Fe2O3–TiO2 Nanocomposites: Influence of TiO2 Content on Their Structure and Activity
Source: Molecules. 2025 Nov 5;30(21):4309. doi: 10.3390/molecules30214309 (PMC12609385; doi:10.3390/molecules30214309)
Supplement: Supplementary file 1 [file molecules-30-04309-s001.zip › molecules-3919647-supplementary.pdf]

Supplementary Materials

Photocatalytic Evaluation of Fe_2O_3–TiO_2 Nanocomposites: Influence of TiO_2 Content on Their Structure and Activity

Israel Águila-Martínez [1], Pablo Eduardo Cardoso-Avila [2], Isaac Zarazúa [1], Héctor Pérez Ladrón de Guevara [1], José Antonio Pérez-Tavares [1], Efrén González-Aguiñaga [1,†] and Rita Patakfalvi [1,*]

[1] Centro Universitario de los Lagos, Universidad de Guadalajara, Lagos de Moreno 47460, Jalisco, Mexico; ing.bioq.iam@gmail.com (I.Á.-M.); isaac.zarazua@academicos.udg.mx (I.Z.); hector.pladrondeguevara@academicos.udg.mx (H.P.L.d.G.); jose.perez5161@academicos.udg.mx (J.A.P.-T.); egonzaleza@enes.unam.mx (E.G.-A.)
[2] Centro de Investigaciones en Óptica, A.C., León 37150, Guanajuato, Mexico; pecardoso@cio.mx
* Correspondence: rita.patakfalvi@academicos.udg.mx
† Current address: Escuela Nacional de Estudios Superiores Unidad León, Universidad Nacional Autónoma de México, León 37684, Guanajuato, Mexico.

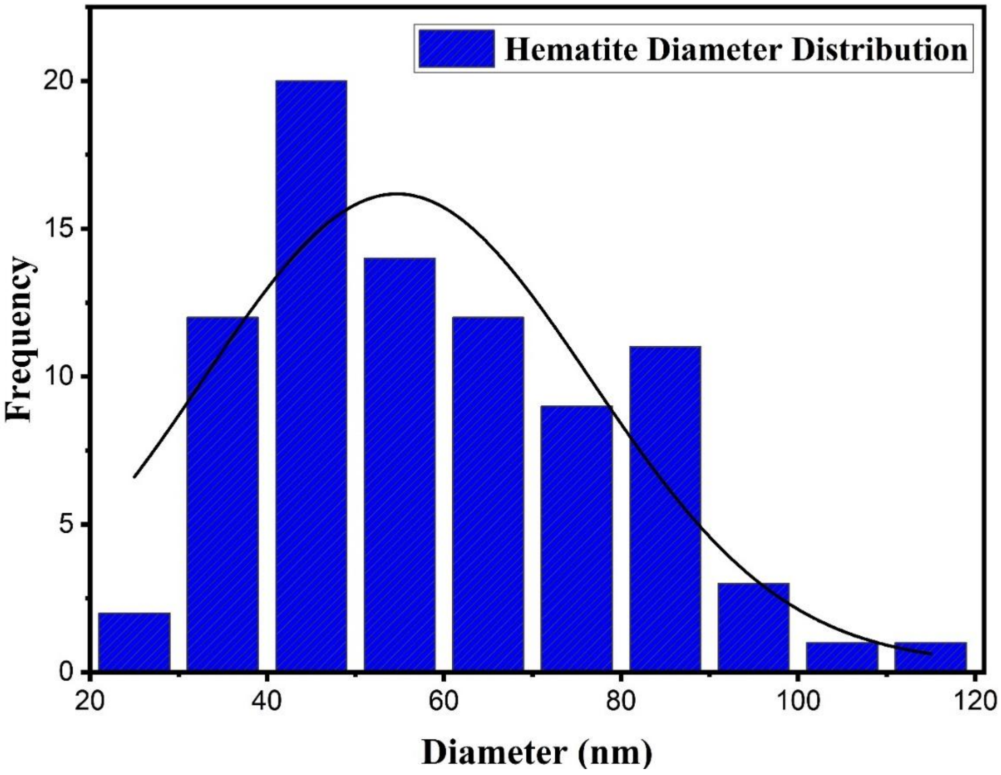

Figure S1. Particle size distribution of hematite nanoparticles (H700). Average size D = 54.7 ± 4 nm.

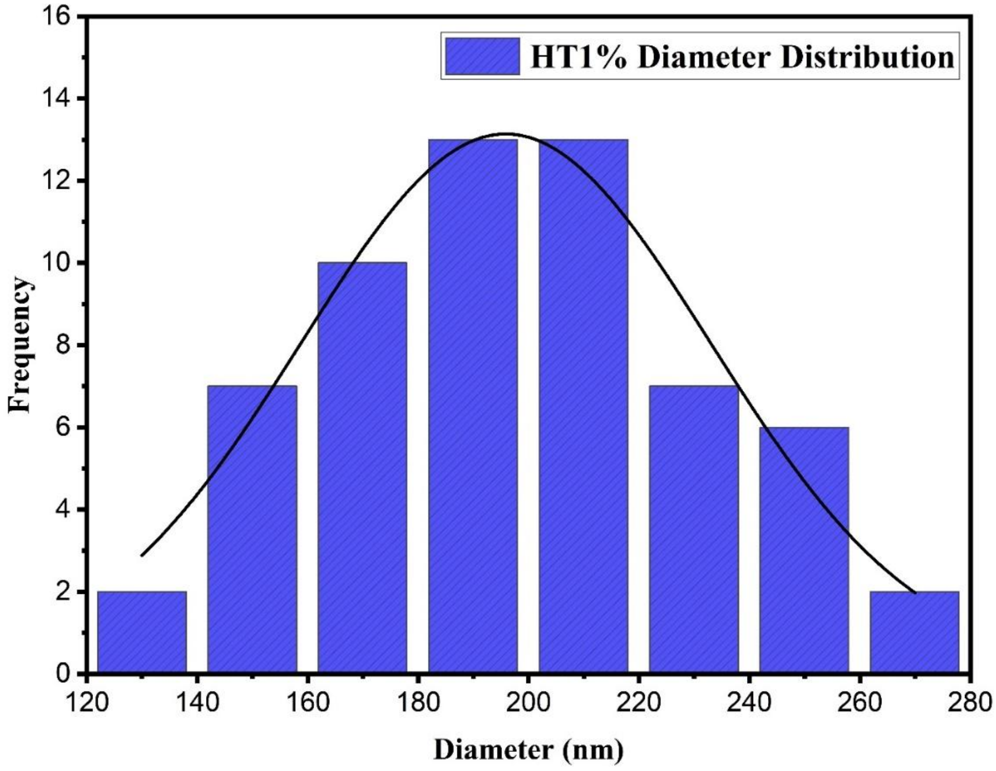

Figure S2. Particle size distribution of HT1%. Average size D = 195 ± 2.9 nm.

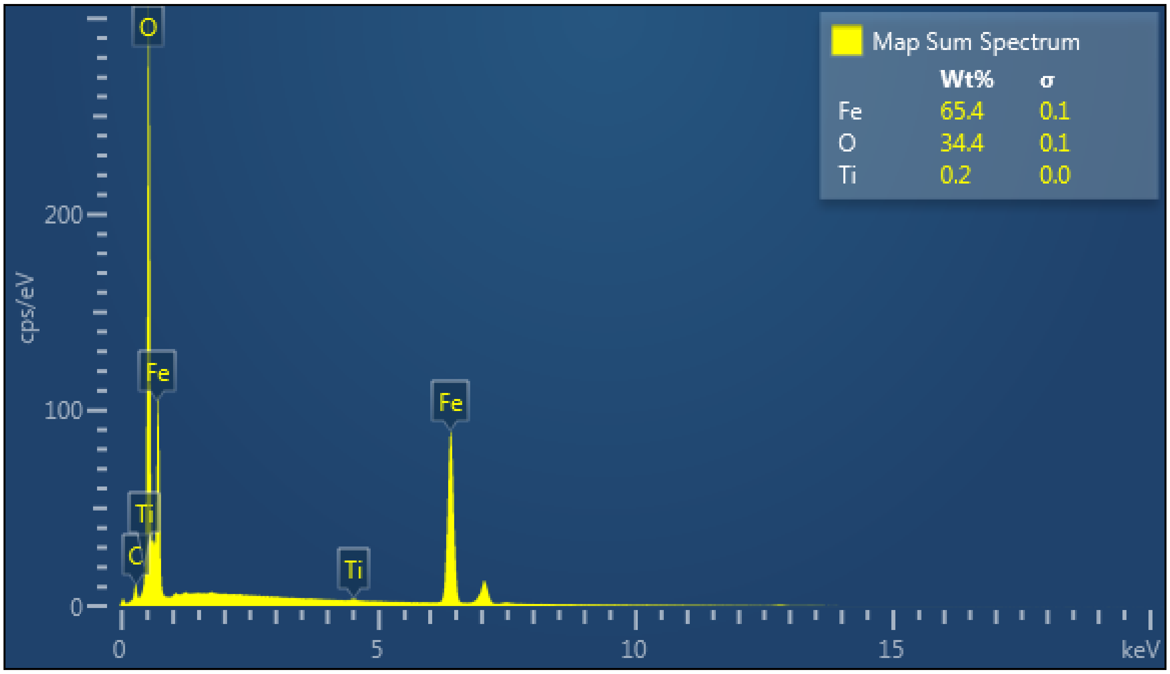

Figure S3. EDS spectrum of the HT1% sample showing the elemental composition.

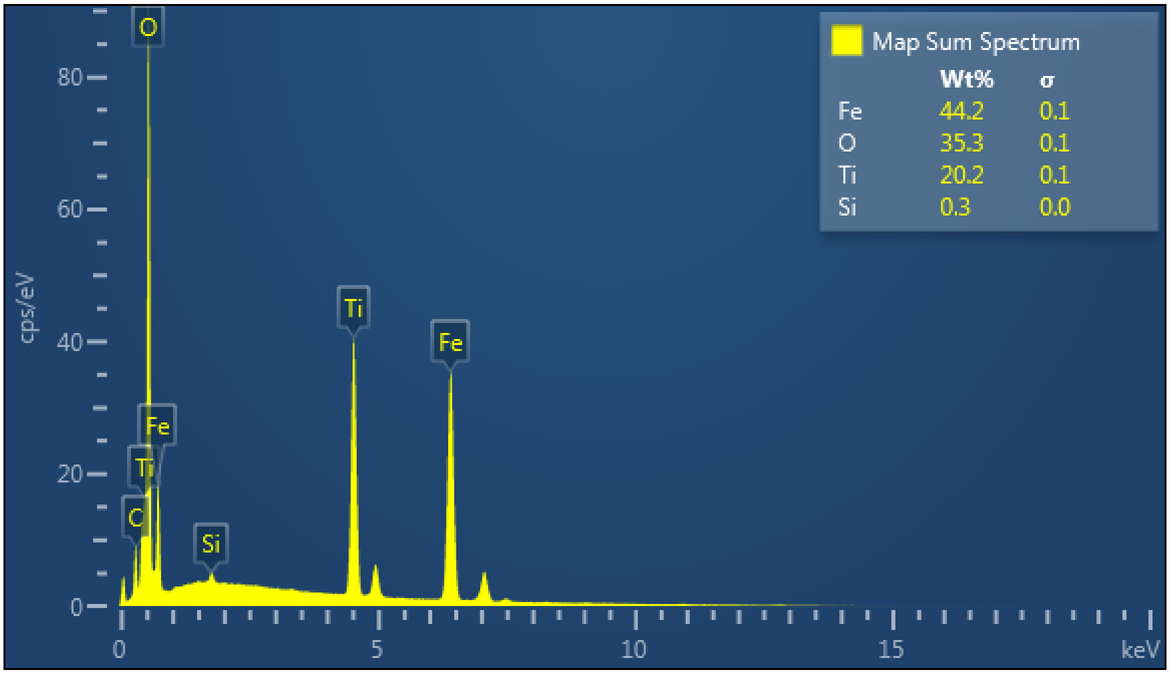

Figure S4. EDS spectrum of the HT50% sample showing the elemental composition.

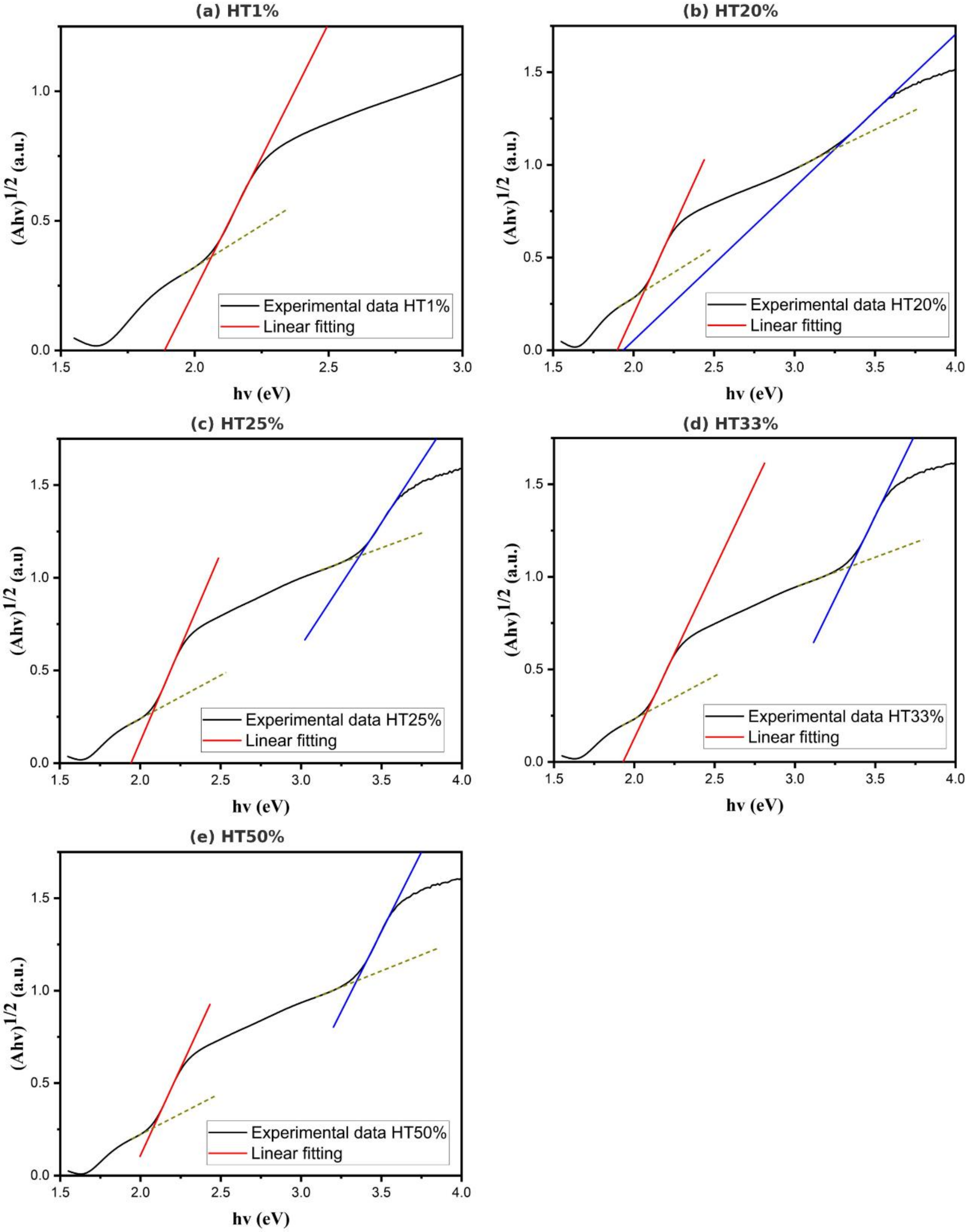

Figure S5. Tauc plots obtained from UV–Vis diffuse absorbance spectra of the Fe_2O_3–TiO_2 binary composites: (a) HT1%, (b) HT20%, (c) HT25%, (d) HT33%, and (e) HT50%. The optical band gap values were estimated by extrapolating the linear fitting of the absorption edge to a defined baseline rather than to the photon energy axis, in order to avoid underestimation due to sub-band gap absorption.

(a)

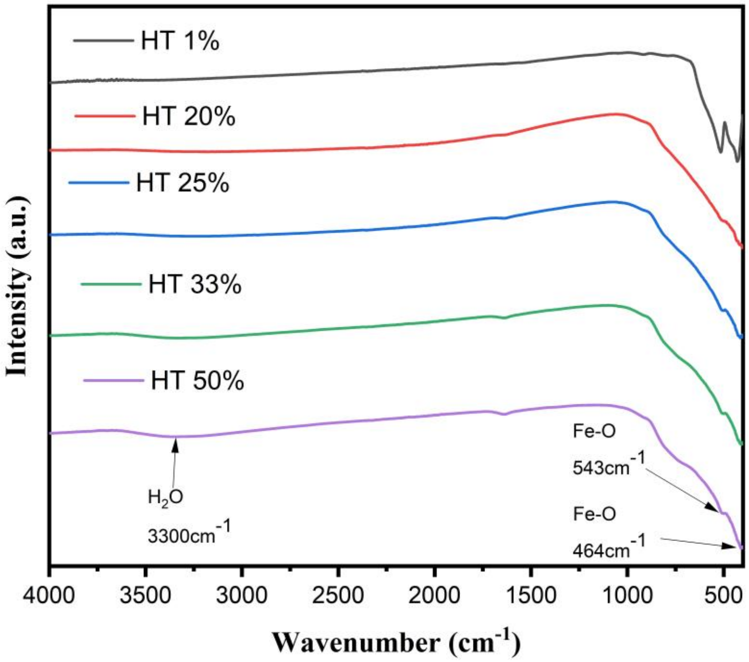

(b)

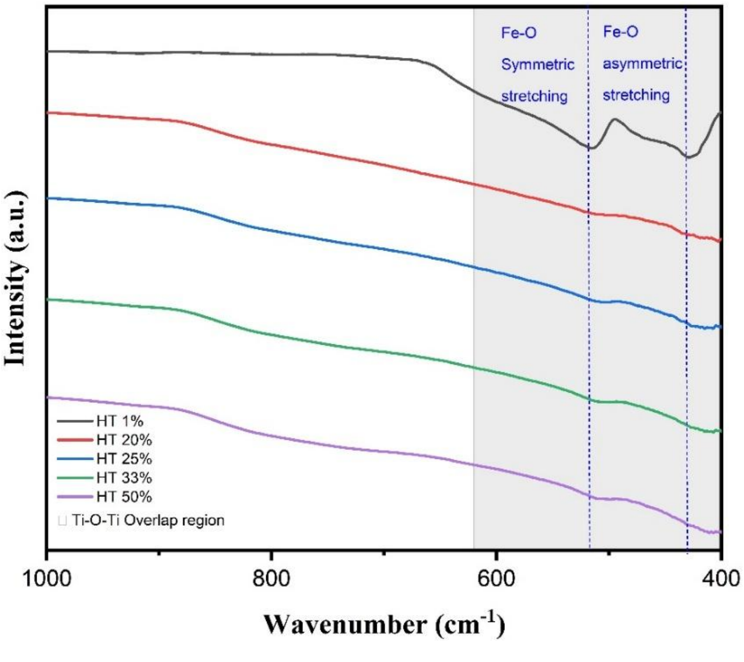

Figure S6. (a) FTIR spectra of Fe_2O_3–TiO_2 composites with increasing TiO_2 content and **(b)** the magnification of the 1000-400 cm^{-1} region. The main Fe–O stretching vibrations are visible around 543 and 464 cm^{-1}.

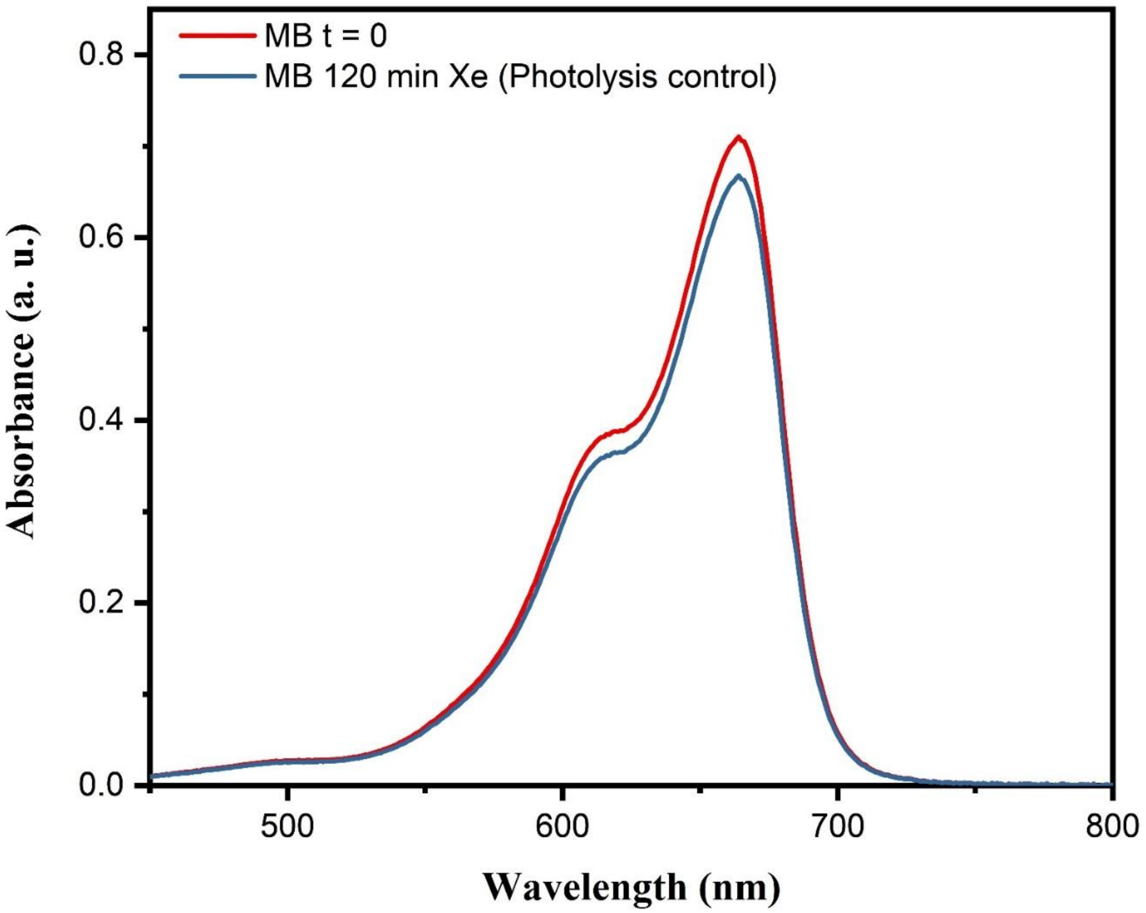

Figure S7. UV–Vis spectra of methylene blue (10 μM) before and after 120 min Xe irradiation (photolysis control). The absorbance decreased by ≈ 6 % at λ_{max} = 664 nm, indicating minor self-photolysis under AM 1.5 G equivalent illumination.

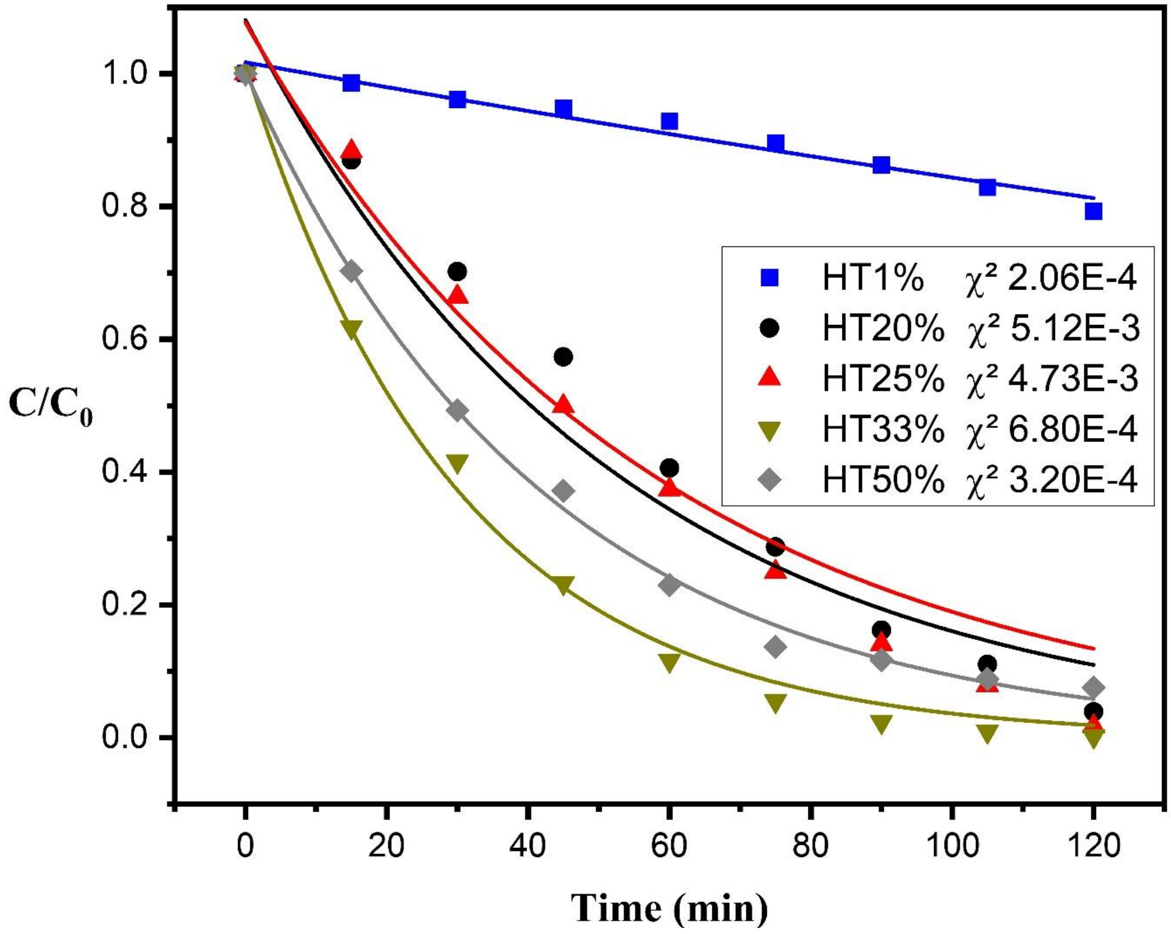

Figure S8. Non-linear pseudo-first-order fitting of methylene blue photodegradation kinetics for Fe_2O_3–TiO_2 composites. Experimental data (symbols) are well described by exponential decay models (solid lines), yielding reduced chi-square (χ^2) values between 2.06×10^{-4} and 5.12×10^{-3}, confirming the suitability of the model. Among all samples, HT33% showed the highest photocatalytic efficiency.

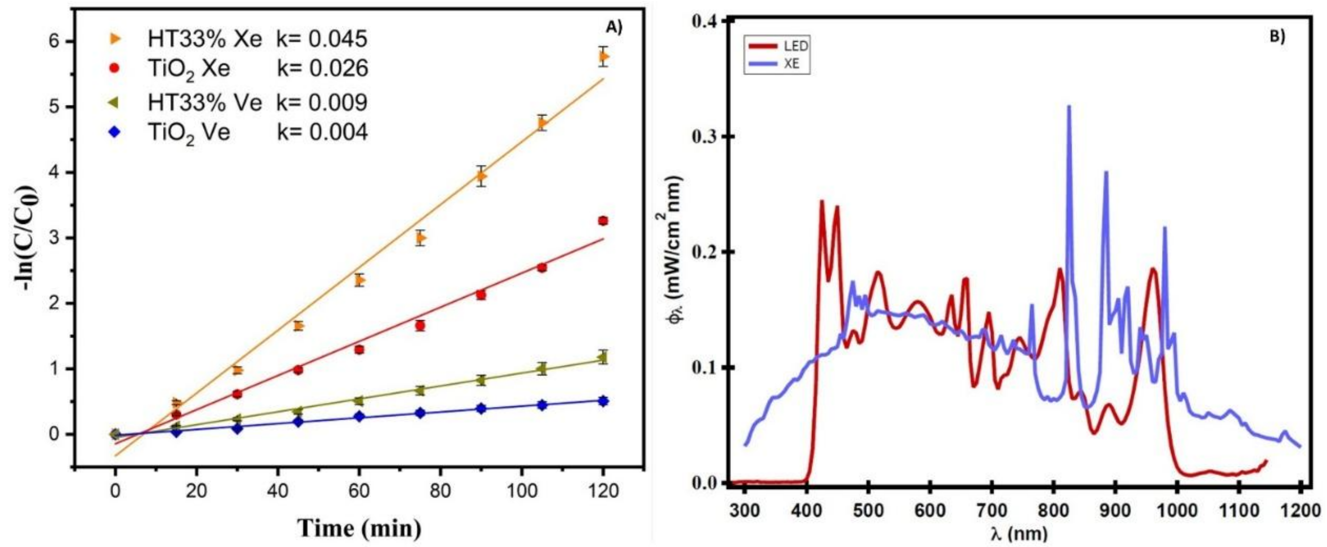

Figure S9. (A) Pseudo-first-order kinetics (−ln(C/C_0) vs. time) for TiO_2 and HT33% composites under xenon (Xe) and UV-free Verasol LED (Ve) illumination, both adjusted to 1 Sun (100 mW cm^{-2}). Apparent rate constants (k, min^{-1}) were obtained from linear fits of the experimental data. Error bars represent the standard deviations from duplicate measurements. (B) Spectral irradiance distribution (ϕλ) of the xenon and Verasol sources. The xenon lamp covers the UV–visible–NIR region (300–1100 nm), whereas the Verasol LED emits exclusively in the visible range (λ > 400 nm). The lower photocatalytic rates observed under visible-only conditions confirm that HT33% retains measurable activity in the absence of UV photons. Figure S11: FTIR spectra of HT33% composite before and after the reusability cycles, showing the characteristic Fe–O stretching bands at 543 and 464 cm^{-1}. The spectra remain very similar, indicating high structural stability after repeated photocatalytic use.

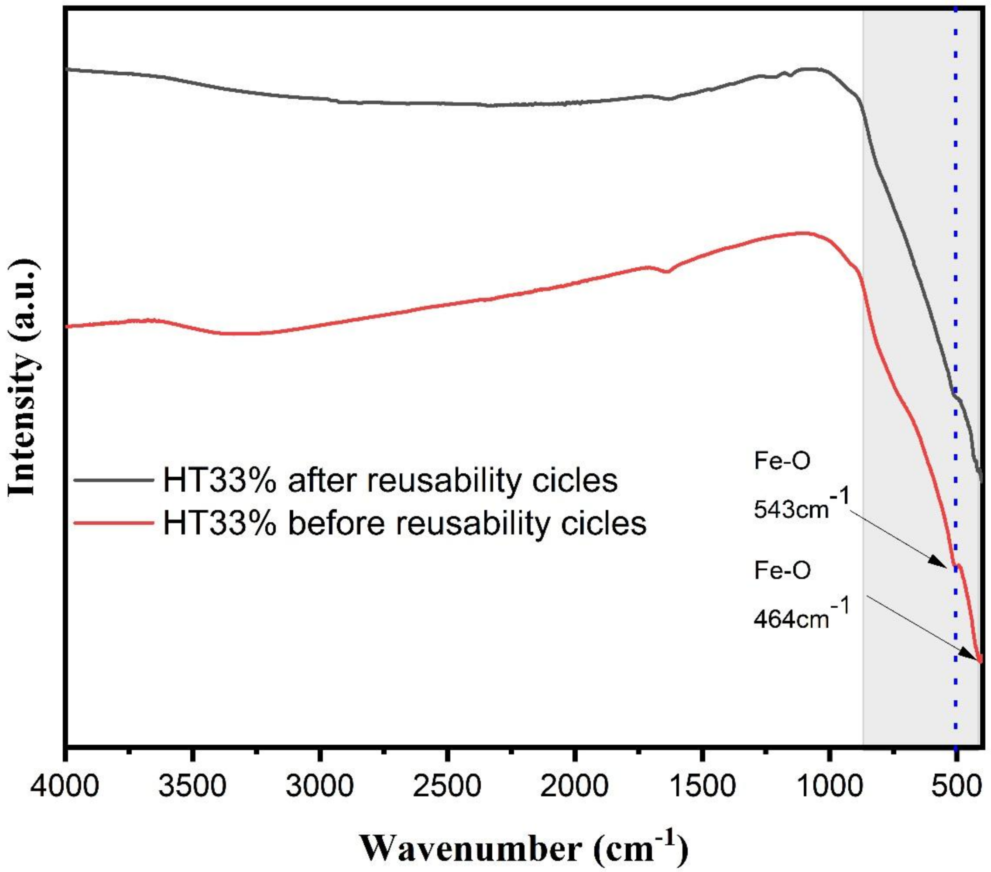

Figure S10. FTIR spectra of HT33% composite before and after the reusability cycles, showing the characteristic Fe–O stretching bands at 543 and 464 cm^{-1}. The spectra remain very similar, indicating high structural stability after repeated photocatalytic use.
